# Progress and Prospects of Nanocellulose-Based Membranes for Desalination and Water Treatment

**DOI:** 10.3390/membranes12050462

**Published:** 2022-04-25

**Authors:** Asif Saud, Haleema Saleem, Syed Javaid Zaidi

**Affiliations:** 1 Center for Advanced Materials, Qatar University, Doha P.O. Box 2713, Qatar; asifsaud111@gmail.com (A.S.); haleema.saleem@qu.edu.qa (H.S.); 2 Industrial Chemistry, Department of Chemistry, Aligarh Muslim University, Aligarh 202002, India

**Keywords:** nanocellulose, membranes, seawater desalination, reverse osmosis, water treatment

## Abstract

Membrane-based desalination has proved to be the best solution for solving the water shortage issues globally. Membranes are extremely beneficial in the effective recovery of clean water from contaminated water sources, however, the durability as well as the separation efficiency of the membranes are restricted by the type of membrane materials/additives used in the preparation processes. Nanocellulose is one of the most promising green materials for nanocomposite preparation due to its biodegradability, renewability, abundance, easy modification, and exceptional mechanical properties. This nanocellulose has been used in membrane development for desalination application in the recent past. The study discusses the application of membranes based on different nanocellulose forms such as cellulose nanocrystals, cellulose nanofibrils, and bacterial nanocellulose for water desalination applications such as nanofiltration, reverse osmosis, pervaporation, forward osmosis, and membrane distillation. From the analysis of studies, it was confirmed that the nanocellulose-based membranes are effective in the desalination application. The chemical modification of nanocellulose can definitely improve the surface affinity as well as the reactivity of membranes for the efficient separation of specific contaminants/ions.

## 1. Introduction

The growing global population together with the natural water resource depletion can increase the freshwater requirement seven-fold [1]. During the upcoming thirty years, it is predicted that the total world population can increase by more than 40 percent and, moreover, the need for domestic, agricultural, and industrial water sources will be extended, predominantly in the developing countries where the water requirement is comparatively higher with respect to its population. The World Water Council reported that by 2030, there would be an extraordinary possibility that almost 3900 million people would reside in water scarce areas. The problem of lack of water is not just an issue of appropriate techniques, but also it is a social and educational issue depending on the country, together with global activities and on technical solutions [2]. For overcoming the problem of water shortages and the greater requirement for clean water, there is a necessity for fresh water sources together with preservation of existing water resources by means of a proper technique for water treatment. Desalination has already proved to be an excellent technology capable of developing the natural hydrological cycle by enriching it with water from oceans and several brackish resources. Desalination is the process of removing excess salts and minerals from saline water to make it fresh water. In water desalination, various technologies have been employed such as thermal-based and membrane-based technologies [1].

Agricultural discharge from saline-alkali lands and the waste generated from pharmaceutical, agri-food, oil and gas, chemical, and aquaculture based industries are the main sources of waste water salinity [3,4] and these sources determine the concentration and composition of the saline water [5]. When the inorganic salt content ranges from 1 to 3.6% *w/w*, then water is considered to be hypersaline, while seawater generally contains 3.5% *w/w* sodium chloride [6]. Wastewater’s hypersaline nature causes major pollution and creates various problems in different ecosystems [7]. Treatment of saline wastewater has become a major concern in many countries. Physical and chemical approaches are typically used to treat saline wastewaters. Traditional procedures, on the other hand, face issues such as increased treatment costs and management of byproducts generated [8]. Recently, several advanced techniques using improved materials have been studied for effective water treatment [9,10].

Membrane-based desalination applications for commercial water treatment plants have acquired a lot of momentum in recent years, [11] especially in industrialized countries [12]. This is due to the fact that these systems are more convenient and effective than chemical treatment plants. The different desalination technologies with the required pressures and membrane pore size are presented in Table 1. Unlike chemical treatment, the membrane filtration systems create safer water [13], thus making them more suitable for various filtration applications [14]. Membranes play an important role in the membrane-based desalination system [15], as they are responsible for the permeation of specific components [16]. As a result, the membrane properties should be well considered while selecting a membrane for the desalination application [17,18]. The membrane material’s surface characteristics (hydrophobicity, pore size, reactive functional groups and surface charge) can influence the efficiency of the desalination system [19,20,21,22,23,24].

**Figure 1:**
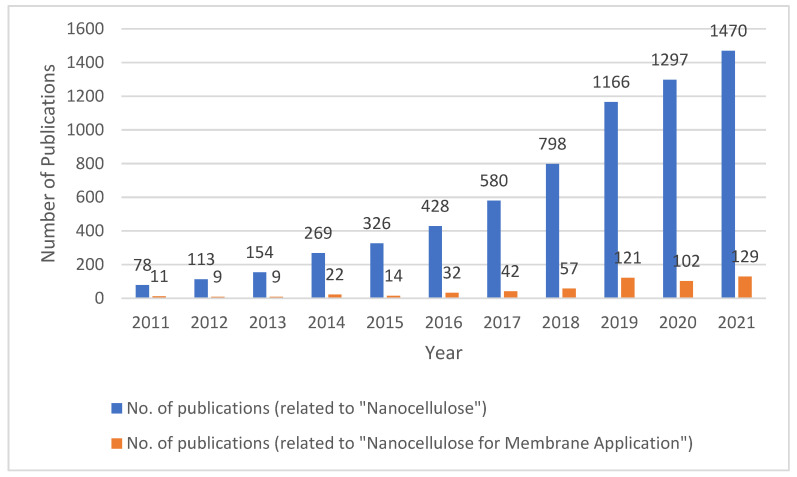
The number of publications (by year) in which the expressions “nanocellulose” and “nanocellulose + membrane” were seen in the title, keywords, or abstract in the last 10 years. These data were obtained from the database scopus.com (24 February 2022).

In order to enhance the physiochemical properties of the membranes, various nanomaterials have been used in the past few years [31,32,33,34]. The nanomaterials can be coated, grafted, or implanted in different layers of the membrane to enhance the physiochemical and performance ability of the membrane. However, some nanomaterials have been reported to be toxic to the environment and harmful to human health [35,36,37,38,39] and this necessitated the development of a renewable, low-cost, and environmentally friendly nanomaterial for the desalination application. The different cellulose based nanomaterials such as cellulose nanocrystals(CNCs), cellulose nanofibrils (CNFs), and bacterial nanocelluloses (BNCs) [40,41,42,43,44] are ideal candidates for addressing the challenges in the field of water desalination. Most of the membranes used for water treatment application have been fabricated from cellulose or non-cellulose organic polymers such as polypropylene, polysulfone (PSF), polyvinylidene fluoride (PVDF), [45] and polyethersulfone (PES) [46]. The nanocelluloses (NCs) are developed from renewable and sustainable substances that are hydrophilic, non-toxic, and less harmful to the environment [47]. Cellulose, the most common natural polymer, has a wide application (Figure 1) in different fields and has a significant impact on the planet in various ways. The advancement of this material in the field of instrumentation and processing technology allowed researchers from different fields to work on the nanoscale structure of cellulose. Ref. [48] The possibility of converting cellulose into nanoscale materials using various methods has opened up a new arena to investigate the properties and possible applications in wastewater treatment [49]. The nanocelluloses are chosen over micro-sized materials for successful desalination, adsorption, and pollutant removal due to their high surface area, nano dimensions, good strength, and non-toxic nature [50,51,52,53]. The wastewater might be hypersaline and also include metals, organic wastes, emulsions, organic and inorganic dyes, and even harmful microorganisms. In recent years, nanotechnology has introduced a number of innovative nanocellulose-based materials that have potential to resonate with the findings in the field of waste water treatment [54,55,56]. The increase in the research interest for nanocellulose can be seen in the number of associated studies, as shown in Figure 1. The figure was created using the scopus.com database. The figure presents the number of publications each year for the past ten years, where the expressions “nanocellulose” or “membrane” were seen in the title, abstract, or keywords of the papers published during the last ten years. The database showed 7092 results for “nanocellulose” and 590 results for “nanocellulose for membrane application”. From the graph, it is evident that there has been clear growth in the interest for nanocellulose for membrane-based application in the past ten years.

To the best of our knowledge, there is no study reported on the application of membranes based on different nanocellulose forms such as cellulose nanocrystals, cellulose nanofibrils, and bacterial nanocellulose for water desalination applications such as nanofiltration, reverse osmosis, pervaporation, forward osmosis, and membrane distillation. We begin this paper by explaining the structure and properties of nanocellulose. Then, we discuss the different applications of cellulose nanocrystals in membrane desalination technologies such as nanofiltration, reverse osmosis, and pervaporation. The next section discusses the different applications of cellulose nanofibers in membrane desalination technologies such as ultrafiltration, nanofiltration, and reverse osmosis. Subsequently, the applications of bacterial nanocellulose have been detailed, along with the outlook of nanocellulose.

## 2. Structure and Properties of Nanocellulose

**Figure 2:**
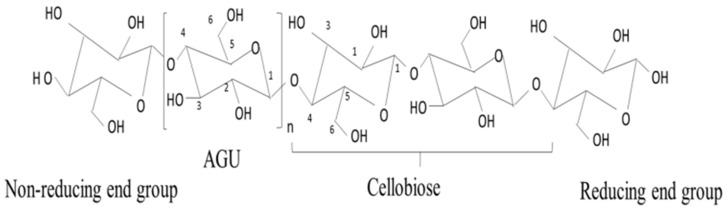
Structural representation of cellulose polymer [58].

Cellulose is a polysaccharide made up of 1,4-D-pyran-type dehydrated polydextrose [57]. Figure 2 presents the structural representation of cellulose polymer. The β-1,4-linkages in the cellulose connect linear D-glucopyranose units through intermolecular hydrogen bonding and hydrophobic interactions, while individual polymer chains give rise to fibrous structure. Each cellulose chain has directional disorderness due to the presence of terminal groups. A hemiacetal group exists at the reducing end of the cellulose chain while a hydroxyl group hangs from the non-reducing end.

Each molecule of cellulose contains three alcoholic hydroxyl groups, and these groups can be chemically altered by different chemical reactions (polymer grafting, esterification acetylation, oxidation, and many more) [59]. Chemical alteration can take place in both heterogeneous and homogeneous environments; since cellulose’s strong crystallinity indicates that it can only be dissolved in a few solvents [60], many alterations have been carried out in a heterogeneous environment [61].

The sources of origin for cellulose are algae, tunicates, bacteria, and some sea animals that are also capable of producing cellulose in abundant quantity [41]. This indicates that cellulose is made up of a mixture of largely crystalline (well ordered) and amorphous (disordered) areas, and these areas, when chemically or mechanically treated, will give rise to different forms of nanocellulose, which will be discussed in this review study.

**Figure 3:**
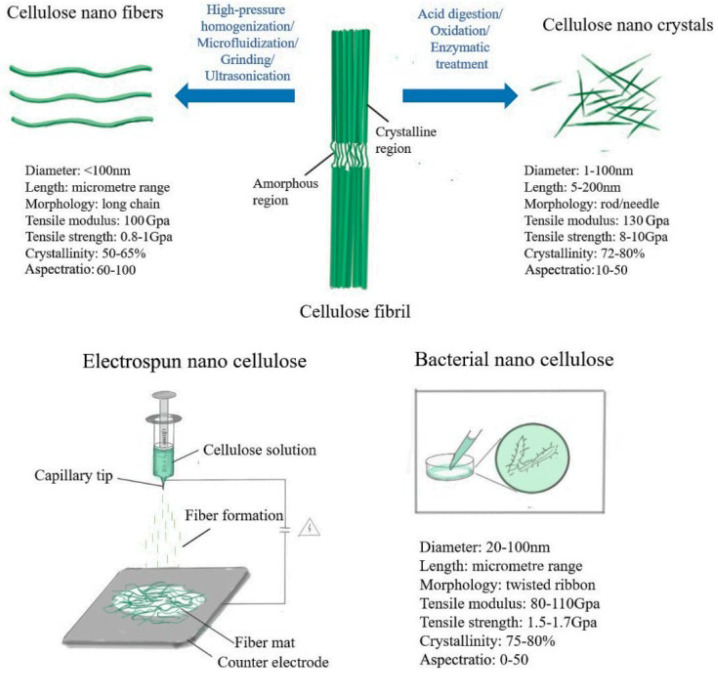
Properties, structure, and synthesis of different types of nanocelluloses [62].

The term “nanocellulose” refers to cellulose that has been nanostructured. The properties which make nanocellulose incredible are that they are harmless, renewable, and degradable, and these make nanocellulose currently useful in several advanced materials [62,63,64]. Nanocellulose has a crystalline or fibrous nature that can be chemically and physically treated [65], and the different types of nanocellulose include cellulose nanocrystals, cellulose nanofibers, and bacterial nanocellulose, as presented in Figure 3 [66]. The BNCs are bacteria-produced nano-structured celluloses. All the different types of celluloses are classified on the basis of their morphology and sources of extraction. The main difference between BNCs and other nanocellulose materials is that BNCs are prepared using a bottom-up approach, which involves bacterial cultivation in an aqueous culture media, whereas CNCs and CNFs can be made using a top-down approach, which involves chemical and mechanical treatments or a combination of mechanical, chemical, and enzymatic processes [67]. Due to the unique properties, nanocelluloses are being used in various waste treatment techniques [67,68,69,70], and one of the most important areas where nanocelluloses are in higher demand is in membrane desalination technology. The incorporation of nanocellulose within the existing membrane can lead to an improvement in the performance of the membranes [32]. The inclusion of nanocelluloses during the fabrication of the membrane can result in good selective permeability, enhanced salt rejection, increased water flux, etc. [71].

## 3. Sources for Obtaining Nanocellulose

In general, the cellulose must be extracted from a source prior to the production of nanocellulose. To date, cellulose has been attained from an extensive range of sources such as plants, bacteria, algae, and tunicates. The properties of CNC are dependent on the source, and therefore appropriate cellulose source material and suitable methods for extraction should be recognized depending on the final properties needed as well as the applications of nanocellulose.

Due to its environmental advantages and the multifunctional nature of nanocellulose, several scientists have begun to examine the capability for producing nanocellulose by using microbial hosts. Just as in algae-based cellulose, bacterial cellulose (BC) has high purity due to the fact that it has no other polymers or functional groups apart from alcohol. Further, nanocellulose generated from bacterial cellulose has improved crystallinity as compared to the plant-derived nanocellulose (Jonas et al., 1998). Cellulose is mostly generated from agricultural residues or by-products (sugarcane bagasse, rice husk, and corn), non-wood lignocellulose (cotton, kenaf, ramie, jute, flax, and hemp), and wood of forest resources. These plant sources for cellulose are plentiful globally and are easily accessible, almost vast, and inexpensively sourced, leading to a lower production cost for cellulose nanocrystals. The cellulose fibers form plants are one of the most widely researched and employed major sources for the production of nanocellulose. Generally, the plant-based cellulose is chosen over bacterial cellulose and tunicate-based cellulose when very thin nanofibers are needed for specific applications. The commonly seen type of plant fiber employed for the preparation of cellulose is wood pulps because of its comparatively higher purity and durability of cellulose, as well as ductile groups and better physical characteristics relative to other plant-based cellulose sources.

Significant advancements towards ecological sustainability have been achieved by employing the forthcoming algae, the underground weapon of green energy. The green alga (Cladophora) is a good source for the preparation of nanocellulose production. The application of the green filament algae for the generation of nanocellulose is interesting, as it could support resolving the challenges related to the pollution of water in coastal areas. In a study carried out by Mihranyan and group [72], the team studies the extremely crystalline properties of Cladophora cellulose, and it was suggested that the extreme inertness of the cellulose reduced the exposure to chemical treatments relative to cellulose developed from conventional plant-based sources.

Furthermore, nanocellulose is also generated from tunicates (invertebrate animals in sea). Several researchers have recognized the application of sea squirts specifically as a current substitute for the preparation of nanocellulose. Nechyporchuk and group [73] has prepared an extremely effective filter membrane to separate oil/water by con-solidating extremely hydrophilic tunicate CNC and cholesteric liquid crystal structure.

## 4. Methods for Obtaining Nanocellulose

Before the production of nanocellulose, most cellulose should be isolated from untreated cellulose pulp that consists of lignin and hemicelluloses. Additional treating of the refined cellulose pulp is necessary for producing CNC/CNF. Generally, the majority CNFs and CNCs are generated by breaking down the cellulose fibers into nanosized fragments (by top–down approach), except for electrospun CNF and bacterial cellulose that employ the electrospinning method (bottom–up approach) and bacteria, respectively. In general, the manufacturing methods use different processes sequentially for producing different kinds of CNC/CNF which differ with respect to surface chemistry, crystallinity, and morphology [74]. The processes include chemical pretreatment, biological treatment, and mechanical disintegration. The appropriate methods applied for producing the nanocellulose from purified cellulose are examined in brief in the following section.

In general, it was noted that mechanical treatment is the main treatment used for producing the CNFs, whereas it is also employed as the post-treatment as well as purification step to produce CNC. Mechanical disintegration is frequently employed for breaking the cellulose pulp into small particles. Nevertheless, effective mechanical disintegration of cellulosic fibers commonly needs simple fibril delamination of cellulosic fiber rather than simply fiber shredding. In a study performed by Park and team [75], the team studied the chemical composition in wood-based CNF and its impact on the defibrillation effectiveness in wet disk-milling, which enhanced defibrillation in the non-existence of hemicellulose as well as lignin in cellulose.

In a research work by Fan and group [76], it was noted that the application of chemical agents is a major contributor to the defibrillation process during the fabrication of nanocellulose. A commonly seen method for extracting CNC from cellulose is acid hydrolysis. Different from mechanical disintegration, the chemical treatment method breaks the amorphous region in microfibrils, putting away the crystalline areas undamaged. The application of green solvents as both solvent as well as catalyst for cellulose hydrolysis has turned out to be a standard technique for the extraction of nanocellulose.

In nanocellulose production, the enzymatic hydrolysis is a frequently utilized method prior to mechanical disintegration of cellulose. Different enzymes have been employed to prepare nanocellulose: ligninases, xylanases, pectinases, and cellulases. A study by Nechyporchuk and team [74] stated that the rheological characteristic of CNF suspension prepared from the enzymatic pretreatment demonstrated excellent flocculation ability relative to CNF developed from chemical pretreatment.

## 5. Application of Cellulose Nanocrystals in Desalination Application

**Figure 4:**
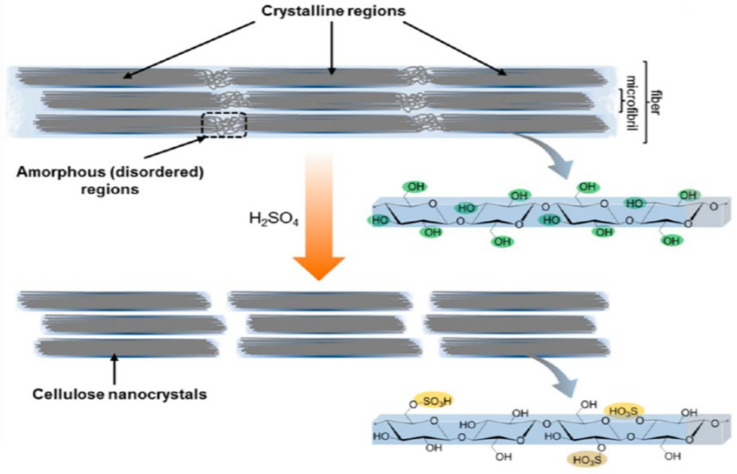
Schematic representation of crystalline region and amorphous region in cellulose and the formation of cellulose nanocrystals. Reprinted with permission from Ref. [78]. Copyright 2014 American Chemical Society.

Depending on the source, bulk cellulose contains a mixture of highly ordered, crystalline regions, and some disordered (amorphous) regions in variable amounts [77], as presented in Figure 4.

The highly crystalline areas of the cellulose microfibrils could be extracted only when a combination of mechanical, enzymatic, and chemical process was imposed on the microfibrils. After these stages, the amorphous part of the microfibrils is removed, leaving only cellulose nanocrystals [78]. CNCs are hard rod-like particles with a nearly perfect crystalline structure made of cellulose chain segments. Whiskers, nanoparticles, nanofibers, micro crystallites, and other terms have been used to describe these nanocrystals, but CNC is the most frequently accepted term [79]. These nanocrystals have higher specific strength, modulus, higher surface area, and distinctive liquid crystalline characteristics as compared to bulk cellulose, which contains more amorphous fractions. CNCs have a high surface-to-volume ratio and a lot of hydroxyl groups, thus, these materials are good for different surface functionalization. The type of interactions that a material exhibits with its atmosphere can be varied by putting any chemical functionality on its surface, and this property of CNCs diverts the researcher’s attention. Surface modification can affect membrane performance, and the surface of CNC has many side hydroxyl groups, which allows for chemical modification, and this can affect membrane performance directly. Desalination membrane results can enhance dramatically when high density functional groups are chemically bonded with reactive hydroxyl groups [47]. By implanting chemical modifiers, a highly cross-linked and more permissive and enhanced salt rejection has been observed as a result. Moreover, the improved surface hydrophilicity provided by the hydrophilic and highly functionalized nature of the CNC modified membrane together with its surface smoothness resulted in its higher organic fouling resistance and mechanical strength compared to commercial membranes. In this section, we have reviewed how the nanocellulose crystal inclusion, whether unmodified or modified, has improved the desalination efficiency of the membranes.

### 5.1. Cellulose Nanocrystals Application in Nanofiltration

Nanofiltration is a technique of removing undesirable substances and pollutants with micro or nanoscale dimensions using a semipermeable membrane. Pore sizes in nanofiltration membranes should be between 1 and 10 nm, and on comparing the pore diameters of membrane used in different technologies, it can be noted that nanofiltration membranes have smaller pore diameters than MF and UF membranes but larger pore diameters than RO membranes. Due to environmental and energy concerns, highly permeable nanofiltration membranes are in high demand for water purification (desalination) and soluble particle separation [27,80,81]. Researchers across different fields are working on fabrication and modification of nanofiltration membranes by using different approaches [82,83,84]. However, the chemicals utilized to manufacture NF membranes are not environmentally sustainable. Cellulose, on the other hand, is the most common naturally occurring polymer consisting of highly oriented microfibrils, and it is an ideal material for the preparation of biodegradable NF membranes because it is renewable, biocompatible, and sustainable [85,86,87].

This section of the study features certain recent studies carried out for the preparation and testing of NF membranes developed using cellulose nanocrystal used in the nanofiltration application. Wang and group [88] have developed an NF membrane and, when operating it at 0.6 MPa pressure, the fabricated membrane showed ultra-high permeation flux up to 204 L m^−2^ h^−1^. Furthermore, the efficiency for salt rejection is more than 97% for Na_2_SO_4_, which they claim as the highest obtained result to date. The Donnan and steric effects of the CNC molecules were responsible for the rejection performance of nanofiltration membranes, resulting in the following rejection order: Na_2_SO_4_ (97.7%) > MgSO_4_ (86%) > MgCl_2_ (15.5%) > CaCl_2_ (11%) > NaCl (6.5%). The extremely higher water permeation flux facilitates nanofiltration at lower operational pressure, thereby making it to an energy-saving process. 

Bai and group [89] A group fabricated a membrane by incorporating cellulose nanocrystals into a polyamide nanofiltration membrane (CNC-TFC-Ms), and it was found that the attachment of CNC molecules in the PA layer reduced the pore size. According to the pore size distribution data, the particle size distribution (PSD) curves became narrower, yet the permeate flux and rejection of NaCl both exhibited a positive deviation when compared to pristine membrane. Moreover, according to side potential assessment, the membrane surface negativity decreased, and the rejection rates of over 98.0% and 97.5% for Na_2_SO_4_ and MgSO_4_ have been reported by the group. This confirmed that the CNC modified NF membrane improved the rejection results for the mono and divalent salts. The CNC-TFC-Ms membrane’s NaCl/Na_2_SO_4_ selectivity reached to 60, indicating their distinct divalent/monovalent separation property.

In a study by Xu and group [90], the team modified PA layer of NF membrane by introducing CNC/silver composite to analyze the post fabrication performance of the membrane. The CNC/Ag modified thin film nanocomposite NF membrane showed some good results with respect to pure water permeability as well as rejection rate and, thus, 25.4 L m^−2^ h^−1^bar^−1^ water permeability and Na_2_SO_4_ (99.1%) salt rejection rate was obtained. In addition, the membrane also showed outstanding antifouling performance with humic acid’s flux recovery ratio of 92.6% and antibacterial properties of 99.4% reduction in *E. coli* feasibility.

Bai and group [91] demonstrated modification of membrane by introducing CNCs into NF membranes and comparing their permeability to the unmodified NF membrane. The team discovered that the modified membrane showed a 60% increase in permeability, and this result was obtained by introducing 0.020 wt.% of CNC into the PA layer. It was also observed that the salt rejection performance of the CNC-TFC membrane was outstanding for both mono and divalent salts Na_2_SO_4_ and MgSO_4_ with the results 98.7% and 98.8%, respectively. In addition, the salt rejection rate for monovalent ions was improved by increasing the concentration of CNCs during the modification of NF membrane. The CNC-TFC membranes showed good antifouling property due to the charge on the surface and increased hydrophilicity, as reported by the group. The antifouling ability of the CNC-incorporated thin film composite membranes was examined by the filtration of 500 ppm humic acid. In the first stage, the membranes were compacted, employing distilled water for achieving a stabilized water flux. Subsequently, the feed solution was replaced with humic acid solution, and then the entire CNC-incorporated thin film composite membranes experienced a serious reduction in the water flux. The results of thin film nanocomposite membrane testing results confirmed that the resistance against humic acid fouling of the membranes was enhanced by the inclusion of CNCs into the PA layer. In the second stage, the membranes were filtered for four hours until a stabilized humic acid flux was accomplished, and subsequently, the membranes were carefully cleaned physically using the distilled water. In the third stage, the cleaned CNC-incorporated thin film composite membranes were filtered using distilled water, and the corresponding flux was recorded. Relative to the unmodified membrane, the higher flux recovery ratios (FRR) values of the thin film nanocomposite membranes indicated an improved cleaning efficiency with higher nanocellulose concentration. The flux decline ratio (FDR) and FRR results confirmed that the nanocomposite membranes performed better than the control composite membrane in both cleaning efficiency and humic acid fouling resistance.

**Figure 5:**
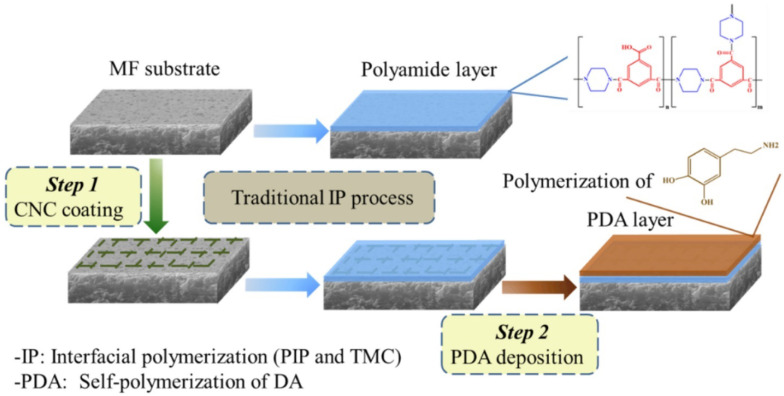
Diagrammatic representation of the CNC-TFC-PDA membrane preparation. Reprinted with permission from Ref. [92]. Copyright 2020 Elsevier B.V. All rights reserved.

In a different research work by Bai and group [92], the team studied TFC modified membranes with cellulose nanocrystal sandwiched layer and a polydopamine (PDA) layer to fabricate the microfiltration substrate (Figure 5). It was found that the CNC interlayer increased membrane electronegativity along with increasing solute permeability and rejection. The modified membranes had a high removal rate for sodium lignosulfonate, congo red, rose Bengal, and alkaline lignin. The salt rejection performance can be explained by the results of the permeability and rejection for MgSO_4_ and CaCl_2_, which was increased by 152% and 8.0% for modified membrane, respectively, when compared to the bare TFC membrane.

Yang and group [93] fabricated polydopamine-modified cellulose nanocrystals incorporated on a thin-film nanocomposite membrane for the application of nanofiltration, showing ultrahigh pure water permeability of 128.4 LMH bar^−1^, superior Congo red rejection of 99.91%, and remarkable salt permeation of 99.33%. Furthermore, on a Congo-red/NaCl mixture, a remarkable salt/dye selectivity of 98% was attained, revealing its enormous potential for successful separation of dye/salt mixtures and recovery of these vital components. Bai and group [94] studied an advanced cellulose nanocrystal incorporated TFN nanofiltration membrane, which showed enhanced water permeability as well as sodium chloride rejection.

All the findings noted from the above-mentioned studies will definitely have a big impact on the future development of high-performance NF membranes for water treatment. The different modifications of the membranes such as the incorporation of pristine CNC, polydopamine-modified cellulose nanocrystals, CNC/silver composite, and cellulose nanocrystal sandwiched layer preparation can definitely improve the performance of membranes in the nanofiltration process.

### 5.2. Cellulose Nanocrystals Application in Reverse Osmosis

**Figure 6:**
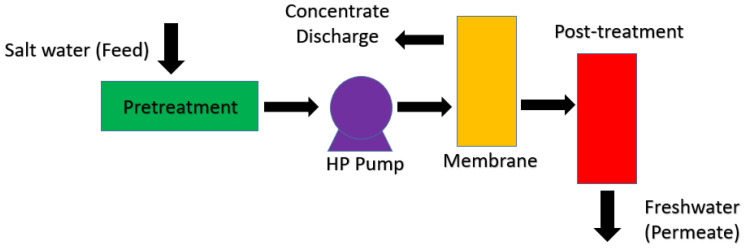
A conventional reverse osmosis system.

Reverse osmosis (RO) is a purification technology that uses a semipermeable membrane to remove ions, hazardous chemicals, and micro and macro particles from contaminated water (Figure 6). An applied external pressure is used in this technology to counteract the osmotic pressure (generated due to concentration difference) [95]. Reverse osmosis desalination systems are widely used around the world because this technology can produce freshwater at the lowest cost [96,97]. The most significant part in RO technology is played by the membranes as the small molecules of water permeate through the membrane pores and generate pure water, while all the contaminants present in water are flushed and the discharged water is termed as reject water. As membranes are semipermeable, salt ions are not permitted to flow across the membrane, and they will be carried within the reject stream [98]. Nanocellulose based membranes could be employed for improving the reverse osmosis performance of membranes for desalination and considerably decreased the desalination cost of USD$5.4 million in 2019 to USD$0.5–1.2 million in Israel, Australia, China, Saudi Arabia, and United States [17].

This section of the study features certain recent studies carried out for the preparation and testing of reverse osmosis membranes developed using cellulose nanocrystal used in the reverse osmosis application. In a study by Liu and group [99], the team demonstrated the incorporation of carbonylated cellulose nanocrystal (CNC_CR_) with active layer (AL-CNC_CR_) and supporting layer (SL-CNC_CR_) of TFC membrane. It was noted that higher permeate flux and salt rejection was obtained in RO testing. The AL-CNC_CR_ membrane showed an excellent pure water flux, and the reported value was 62.17 ± 3.12 LMH. On the other hand, the team reported a conflicting result for the flux for the SL-CNC_CR_ membrane as a decreased value of pure water flux for SL-CNC_CR_ membrane was noted, i.e., 45.02 ± 3.37 LMH. The contrasting result for the pure water flux was due to the difference in position of entrenchment of CNC_CR_ in the TFC membrane. Both modified membranes showed increased value for salt rejection for sulphate ions. The AL-CNC_CR_ rejection ratio increased to approximately 105.2%, and SL-CNC_CR_ membranes showed 107.5% rejection ratio for sulphate ion. The lowest salt rejection value was shown by an unmodified one which was approximately 94.8 ± 0.7% and 89.7 ± 1.5% for Na_2_SO_4_ and MgSO_4_, respectively. Both modified membranes showed good stability under the harsh conditions along with a good water flux and increased permeation. A similar phenomenon as in the case of sulphate ions was also observed in the rejection of sodium chloride; the increased value for chloride ion rejection of SL-CNC_CR_ membrane (134.4%) compared with AL-CNC_CR_ membrane (108.7%) compared with pristine membrane is again due to the implantation location of nanocellulose.

Asempour and group [100] developed a new thin-film nanocomposite membrane with cellulose nanocrystals 0.1% *w/v* loaded in the polyamide active layer. The group observed the membrane performance with the synthetic brackish water (SBW) and the outcome showed that the fabricated membrane demonstrated two times increased water flux from 30 to 63 ± 10 L/m^2^·h, without compromising the salt rejection, which is 97.8%. To evaluate the fouling properties, the team used BSA (Bovine Serum Albumin) in the feed solution with a stable concentration of 300 ppm, and good fouling resistance properties were observed for the modified membrane.

Researchers have pointed out that the main disadvantages with the RO filtration membrane are the low rejection rates (metal ion) [101] and an increase in fouling tendency [102]. Due to these reasons, the efficiency of the RO membranes reduces, and the scientists are continuously working to overcome these limitations. Hence, by considering the chemical structure of nanocellulose, many researchers implanted nanocellulose to the membrane just to overcome these issues and project the suitable path for the problem. Park and group [103] developed a poly(acryloyl hydrazide)-grafted cellulose nanocrystal (CNC-PAH) membrane that showed better water permeance, salt rejection, and organic fouling resistance as compared to the commercial membranes. Moreover, it showed higher boron capturing ability and rejection due to the abundance of hydroxyl and amine groups in it. Membrane fouling is influenced by the membrane surface’s structure (roughness), hydrophilicity, and charge density [104]. The reason for fouling is the roughness of surface area and the surface area roughness increases, fouling will increase simultaneously as foulants would have a larger surface area to adhere to [105] and obstructs hydrodynamic rinsing-induced flux recovery by producing foulant valley blockage [106]. Hence, the enhanced antifouling performance and better flux recovery of the poly-acryloyl hydrazide modified membrane can be explained by its smoother surface.

Obtaining cellulose nanocrystals from natural waste made CNC synthesis more economically sound, and one of the reported wastes, i.e., sawdust, is a waste generated by the forestry and timber industries, accounting for more than 40% of all wastes [107]. Most of the sawdust waste is not transformed into valuable goods and is usually disposed of in landfills. Sawdust-derived cellulose nanocrystals embedded in polyamide were created by Adeniyi and group [108], The team discovered high rejection rate values for sodium chloride (98.3 ± 0.8%) and calcium chloride (97.1 ± 0.5%) at specific concentrations of sodium chloride (1500 ppm) and calcium chloride (2500 ppm) solutions. Additionally, in this study, the water flux was greatly increased, more than 23%. The membrane was noted to be extremely hydrophilic and thermally stable.

**Figure 7:**
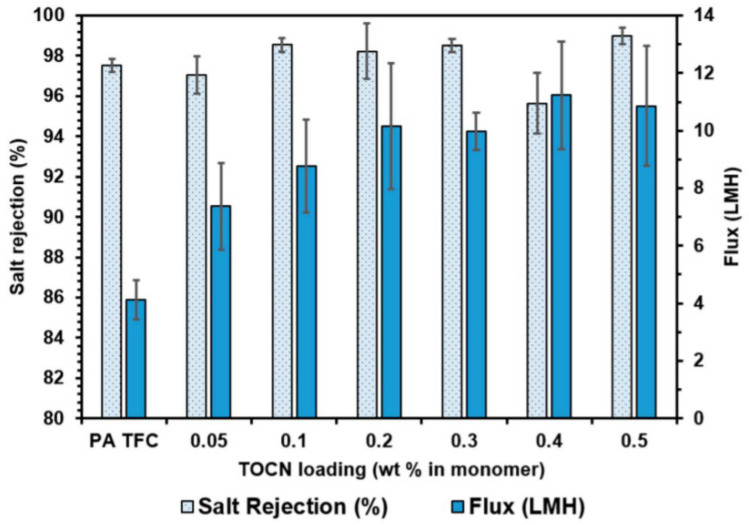
Flux and salt rejection results for TOCN containing TFN membranes fabricated using the monomer dispersion method [108].

In a study by Smith and group [109], the team fabricated 2,2,6,6-Tetramethylpiperidine-1-oxyl (TEMPO)-oxidized cellulose nanocrystals (TOCNs) modified RO thin-film nanocomposite membrane, and the best improvement was obtained by using the monomer dispersion method with 0.5 wt.% TEMPO-oxidized cellulose nanocrystals loading. The increased water flux and salt rejection data provided an evidence of improvement in the membrane performance when compared to the unmodified PA layer of pristine membrane. Water flux for TOCN modified RO membrane was 260% as compared with the unmodified one, and the salt rejection increased to 98.98 ± 0.41% from 97.53 ± 0.31%, as shown in Figure 7. The most important parameter that defines the formation of nanochannels and pore diameter is the strong amalgamation between TEMPO-oxidized CNCs and PA layer. Due to the presence of electronegative atoms, molecules can easily form hydrogen bonds [110], and these hydrogen bonds provide strength and availability of nanochannels, whose dimensions can be altered by providing different conditions. Within this alteration, a significant number of hydrogen bonding reduced the nanochannel dimension, increasing the membrane’s salt rejection ability and resulting in high salt rejection at all loading levels examined in this study.

Therefore, from the analysis of different studies in this section, it was noted that the CNC incorporated membranes can significantly improve the performance of membranes in the reverse osmosis process. The different modifications of the membranes such as the incorporation of pristine CNC, 2,2,6,6-Tetramethylpiperidine-1-oxyl (TEMPO)-oxidized cellulose nanocrystals (TOCNs), carbonylated cellulose nanocrystal, and poly(acryloyl hydrazide)-grafted cellulose nanocrystal (CNC-PAH) membrane can definitely improve the performance of membranes in the reverse osmosis process.

### 5.3. Cellulose Nanocrystals in Pervaporation

The basic membrane system for effectively separating a liquid mixture is pervaporation (PV). Since traditional methods are ineffective in separating mixtures, PV is a new technology that can help in a very efficient separation of liquid mixture [111]. In both the fundamental and applied aspects of PV, significant progress and exciting breakthroughs have been made in recent decades [112]. In PV applications, different studies have focused on the modification of nanocomposite membranes fabricated with nanocellulose [113]. Different researchers have developed nanocomposite membranes with nanocellulose that show outstanding results in terms of selectivity, salt rejection, permeability, [114] and also desirable and effective surface morphology that enhance the membrane efficiency and its mechanical properties [115].

Prihatiningtyas and group [113], by utilizing a solution casting method, fabricated cellulose triacetate/cellulose nanocrystals (3%) (CTA/CNCs) nanocomposite pervaporation membranes. The membrane was modified to a sponge-like structure which offered the evidence for incorporation. After running the modified pervaporation membrane, it was observed that the membrane showed an increment in the water flux with the factor of 3 and it changed from 2.16 kg·m^−2^·h^−1^ to 5.76 kg·m^−2^·h^−1^. The casting blade height was reduced from 200 µm to 100 µm, resulting in a flow of 11.68 kg·m^−2^·h^−1^, and a NaCl rejection was maintained at 99.9%. For 12 h of separation, the CNCs 3%—CTA PV membrane with a casting blade height of 100 µm performed well. Water and NaCl was separated very well with this newly created PV membrane. Furthermore, when compared to a pristine membrane, it had a significantly higher water flux and, hence, might be used for desalination.

The influence of different solvents on the morphology, desalination, and flux performance of cellulose triacetate/cellulose nanocrystals (CTA/CNCs) nanocomposite PV membranes was studied by [116]. The DMSO-based membranes produced a nanocomposite membrane with uniformly distributed CNCs on the membrane surface and a matrix with self-assembled structure. This membrane demonstrated a high-water flux of 11.67 kg m^−2^ h^−1^ and 99.9% NaCl rejection when the feed solution of 30 g L^−1^ of NaCl was used. Furthermore, for a highly saline feed (up to 90 g L^−1^ of NaCl), the produced nanocomposite membranes demonstrated good PV desalination efficacy.

Kamtsikakis and group [117] fabricated a different CNC incorporated nanocomposite membrane and explored the ethanol-recovery pervaporation performance for the mixture sample of ethanol-water. The fabricated membrane with CNCs integration was based on a polystyrene-poly(butadiene)-poly(styrene) (SBS) matrix and was further decorated with oleic acid moieties (OLA-CNCs). The team studied the effect of alteration on pervaporation performance and, as a result, it was discovered that the relative increase in water flux (172%) was significantly higher than the relative increase in ethanol flux (51%) when equated to the separate fluxes through the unmodified SBS. The addition of CNCs to the SBS resulted in a poorer ethanol purity in the permeate (26%) compared to the unmodified membranes (38%).

In a study by Prihatiningtyas and group [118], the team developed a nanocomposite membrane with varying quantities of feed solution and tested its permeability and salt rejection without compromising its selectivity. The team noticed an increase in the CTA/CNCs membrane’s water flux as compared to an unmodified membrane that produced flux of (3.6 kg m^−2^ h^−1^). The team used a 90 g/L NaCl hypersaline solution and ran the experiment for 30 min. They discovered that the water flux increased dramatically, reaching 107.5 kg m^−2^ h^−1^ with more than 99.8% salt rejection. The water flux reduced to 58.5 kg m^−2^ h^−1^ when 200 g/L NaCl was used as the feed solution, and the alkali-treated membrane showed stable performance when evaluated for 90 g/L and 200 g/L NaCl for 12 h.

Rahimi and Kashkoul group [119] incorporated a functionalized cellulose nanocrystal within the TFN’s polyamide thin layer. Thin-film nanocomposite membranes were designated as TFN0.0, TFN-1, TFN-5, and TFN0.1, respectively, based on CNC (wt./v.%) concentrations of 0.01, 0.05, and 0.1. The water contact angle for TFN incorporated with 0.1 wt./v.% of CNC was reduced from 69° for TFN0.0 to 45° for TFN incorporated with 0.1 wt./v.% of CNC. TFN0.05 was the best performing membrane, with a water flux of 21.34 L/m^2^ h. This was roughly 140% greater than TFN0.0 in an identical operational condition. In comparison to a pristine membrane, CNC also decreased the amount of concentration polarization in TFN0.05, which resulted in higher protein fouling resistance and a lower water flux drop.

Hence, from the examination of various studies in this section, it was noted that the CNC incorporated membranes can considerably improve the performance of membranes in the pervaporation process. Table 2 presents the overview of the studies carried out using CNC-based membranes for the desalination and water treatment application. The different modifications of the membranes such as the incorporation of pristine CNC, functionalized cellulose nanocrystal, and cellulose triacetate/cellulose nanocrystals (CTA/CNCs) nanocomposite can enhance the performance of membranes in the pervaporation process.

## 6. Cellulose Nanofibrils (CNF): Structure, Properties, and Desalination Application

Cellulosic nanofibers have been intensively studied among the different nanostructures formed from cellulose due to their outstanding mechanical strength, flexibility, ability to functionalize and integrate, biodegradability, chirality, thermostability, and low thermal expansion. Young’s modulus and tensile strength of CNFs are comparable to Kevlar fibers and are five times that of mild steel [128]. In the axial direction, the CNFs have the same thermal expansion as quartz [129]. CNFs have been proposed as a potential matrix for a wide range of applications, including medical application, energy storage, adsorption, wastewater treatment, biomedical materials, nanofillers, filtration media antimicrobial activity, and membrane modification for desalination.

CNFs can form dense membrane with the help of hydrogen bonding during the water loss process of CNF solution. CNFs can interact with adjacent nanofibers hydroxyl group, and plenty of OH groups become interlocked into colloidal form in aq. solution and generate a CNF self-assembled membrane. The CNFs’ integrated membranes have good strength qualities and lower permeability in comparison with high and low-density polyethylene membrane [130] of similar thickness, and hence these membranes can be used for water desalination application.

CNF’s biocompatibility is a key feature that makes them ideal for use in filtration systems and membranes for water purification and desalination. CNFs are excellent biocompatible materials which makes them suitable for desalination application. Certain precautions are taken for the usage of materials in water filtration systems, such as it should not release and deteriorate harmful elements. The CNF structure’s stability is enhanced by its crystalline character. When combined with other membrane materials having low crystallinity, CNF imparts stiffness and high strength to the nanocomposite membrane, thereby increasing water flux and antifouling in addition to the intrinsic properties [131,132,133,134]. In addition, CNFs are one of the best and cheapest options as they exhibit high-permeability, ultrahigh water flux, better hydrophilicity, and good salt rejection [135] when used in membranes. The hydroxyl groups present on the CNF surface develop powerful hydrogen bonding, which after drying can convert them to extremely degradation resistant. The testing on the biodegradability (EN 14046) of cellulose nanofibril films and papers with cellulose nanofibrils demonstrated that the entire products of cellulose nanofibrils examined were noted to be biodegradable as per the necessities set in the standard EN 13432 [94]. Some outstanding performance has been observed by integrating cellulose nanofibers in the membrane modification, which is discussed in the following section. There are numerous CNF-related experiments reported throughout the years, particularly with respect to the development of CNF incorporated membranes for filtration application. The application of CNFs in membrane production can be divided into numerous categories. The most widely described methods involve coating or infusing functional CNF onto/into a porous scaffold, while others involve including CNF into the composite suspension or using CNF as the major element for inorganics attachment.

### 6.1. Cellulose Nanofibers in Ultrafiltration Application

Ultrafiltration is a dedicated membrane-based filtration technology that improves the pressure-mediated suspension of pathogens as well as solid waste from waste solution. The product water obtained subsequent to the ultrafiltration process is extremely pure and free from any pathogen waste. The utilization of cellulose as well as its derivatives to make membranes for the application in ultrafiltration is well recognized at the industrial level. The usage of CNFs has been studied greater as compared to the CNCs to develop films for UF membranes. This section of the study features certain recent studies performed for the preparation and testing of ultrafiltration membranes developed using cellulose nanocrystal used in the ultrafiltration application.

In a study carried out by Hassan and group [136], utilized high-lignin unbleached neutral sulfite’s cellulose nanofibers for the application in ultrafiltration. The team made a comparison of the developed membrane with the bleached UF membrane on the basis of water flux. During the 2 h of experiment, the team obtained the following data: water flux for bleached membrane was around ~27 L/h/m^2^/MPa, while for unbleached one reached about ~53 L/h/m^2^/MPa. Hence, the water flux efficiency was 96% higher in the case of the unbleached membrane, but when the experiment was run for 1 h, the improvement in flux as a result of using unbleached nanofibers instead of bleached ones was ~120%. The water flux value for the bleached membrane was 4.4 L/h/m^2^/MPa after 30 min of passing the BSA solution (the first BSA cycle), and for the unbleached membrane it was 36.5 L/h/m^2^/MPa.

Soyekwo and group [137] opted for the approach of surface modification of ultrafine cellulose nanofiber (UCN) membranes via interfacial polymerization to make ultrathin polymeric nanofiltration membranes. In this study, the membrane was made up of an ultrathin selective layer interlaced with a cellulose nanofiber matrix. The developed membraned showed good water flux, salt rejection, and resistance to extreme conditions. The results for mono and di valent salt (rejection %) at pH 5.5 were as follows: MgCl_2_—89.7%, MgSO_4_—65.3%, NaCl—43.6%, and Na_2_SO_4_ (39.1%). The team concluded that the membranes showed higher rejections towards divalent cations (Mg^2+^) at pH less than 7 and higher rejections toward divalent anions (SO_4_^2−^) at pH greater than 7. The reason behind displaying lower rejections toward monovalent ions is that pH has an effect on salt flux, and in contrast to rejections, salt fluxes decrease as pH rises. Furthermore, the fouling ratios for reversible (F_r_) and irreversible (F_ir_) fouling were 28.1% and 16.7%, respectively. After cleaning, the recovered pure water flux was restored to 27.3 L m^−2^h^−1^bar^−1^, equating to 83.3% flux recovery ratio with bovine serum albumin (BSA) mixed salt solution. Nanofiber-based UF membranes based on the thin-film nanofibrous composite format having a nanocomposite barrier layer consisting of cross-linked poly(ethylene glycol) matrix as well as ultra-fine CNFs were examined in a study by Wang and group [138]. It was noted that the membrane developed was extremely hydrophilic and possessed superior antifouling characteristics, which were proved by long-term and short-term fouling experiments using bovine serum albumin (BSA) solution (1 g/L).

Therefore, from the analysis of different research works in this section, it was confirmed that the CNF-embedded membranes can considerably improve the performance of membranes in the ultrafiltration process. The different modified membranes such as the thin-film nanofibrous composite format having a nanocomposite barrier layer consisting of cross-linked poly(ethylene glycol) matrix as well as ultra-fine CNFs, surface modified ultrafine cellulose nanofiber (UCN) membranes, and high-lignin unbleached neutral sulfite cellulose nanofiber-based membranes can enhance the performance of membranes in the ultrafiltration process.

### 6.2. Cellulose Nanofibers in Nanofiltration

**Figure 8:**
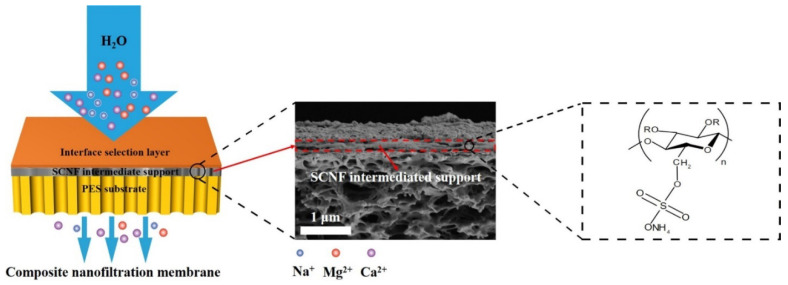
Novel composite nanofiltration membrane using sulfated cellulose nanofibril (SCNF) for nanofiltration [139].

Nanofiltration (NF) membranes are of greater interest in the desalination of brackish water and the purification of drinking water. Improvement in the separation performance as well as permeation still remain to be a great challenge in the latest innovative NF membranes. The nanofiltration process happening at lower pressure is in huge demand as it could save energy, and it could be highly conceivable in the case that the membrane could generate excellent permeation flux. Mohammed and group [139] fabricated reduced graphene oxide/cellulose nanofibers to test the nanocellulose fabricated membrane’s performance on various parameters of nanofiltration. The thick stacking of reduced graphene oxide frequently resulted in limited water flux, and that was the reason nanocellulose has been entrenched with the reduced graphene oxide. The obtained results are as follows: pure water permeance of 37.2 L m^−2^ h^−1^ bar^−1^ was obtained by maintaining the rejection above 90% for Acid Fuchsin (1.2 nm), Rose Bengal (1.5 nm), and Brilliant Blue (2.2 nm). With the graphene oxide loading constant (31.83 mg m^−2^), the CNF free graphene membrane exhibited a permeance of 0.33 L m^−2^ h^−1^ bar^−1^ and was steadily improved to 0.9 L m^−2^ h^−1^ bar^−1^ at 1:2 weight ratio. This clearly showed a flux enhancement with the addition of CNFs in the membrane. (Li. W and team [138] fabricated a novel composite nanofiltration membrane (Figure 8) using sulfated cellulose nanofibril (SCNF) for nanofiltration application. The advanced membrane showed higher Na_2_SO_4_ rejection above 98% along with a water permeability higher than 30 L·m^−2^·h^−1^ under two bars.

Moreover, it continues to be a great challenge for developing a superior-performance NF membrane having good antifouling properties as well as higher perm-selectivity. In a study reported by (Xia et al., 2022), the team developed an advanced TFN membrane by in situ incorporation of zwitterionic nanocellulose at the time of interfacial polymerization process. The resulting membrane showed an enhanced water permeance and improved enhanced divalent salt rejection because of the enhanced surface hydrophilicity as well as higher surface area and the super hydrophilic interfacial nanochannels. Under the synergy of zwitterions and nanocellulose, therefore, the membrane developed demonstrated higher perm-selectivity together with beand antifouling properties, which would have promising potentials in separation fields.

Hence, from the examination of different research works in this section, it was proved that the CNF-embedded membranes can noticeably improve the nanofiltration performance of membranes. The different modified membranes such as those of zwitterionic nanocellulose modified membranes and reduced graphene oxide/cellulose nanofiber incorporated membranes could improve the performance of membranes in the ultrafiltration process.

### 6.3. Cellulose Nanofibers in Reverse Osmosis

As it was noted in the previous section, CNFs are noted to have promising application in ultrafiltration and nanofiltration based desalination technologies. From the analysis of different studies, it can be noted that the CNFs are also have having promising application in the reverse osmosis process. Liu and group [82] demonstrated the application of cellulose nanofibers as a cheap and biodegradable material to enhance the efficiency of membranes for RO application. The team fabricated the advanced membrane by interfacial polymerization with 2,2,6,6-tetramethylpiperidine-1-oxyl radical (TEMPO)-oxidized CNFs by integrating it into the polyamide layer of a thin film composite. The optimized loading of CNFs into the TFC membrane enhanced the permeance of the membrane by more than 50% (29.8 L m ^−2^ h ^−1^ at 1.5 MPa) while maintaining high NaCl rejection of 96.2%. The chlorine stability tests revealed that the fabricated membranes outperformed existing TFC membranes in terms of chlorine resistance.

Another modification was shown by Cruz-Silva and group [134], in which the team fabricated and examined the desalination performance of a RO membrane by coupling CNF with an aromatic PA layer. The team found that the membrane became stronger and stiffer as a result of the strong bonding between PA matrix and CNF, and due to this the matrix mobility decreased. Higher water permeability is dependent on the membrane’s hydrophilicity; hence, the fabricated membrane showed an increase in water permeability, indicating the improved hydrophilicity. The CNF-PA membrane showed great hydrophilicity, which is an important parameter for the nanocomposite membrane’s higher water permeability. The antifouling performance and chlorine resistance of the membranes were also improved. The following results were reported subsequent to the study: the CNF membrane showed a diffusion coefficient of 0.37 × 10^−5^ cm^2^ s ^−1^, which was 40% higher than the diffusion coefficient, 0.21 × 10^−5^ cm^2^ s ^−1^, of the pristine PA membrane. The antifouling performance with bovine serum albumin (BSA), water permeability, chlorine resistance, and salt rejection rate of the membranes were also investigated, and it was discovered that after 140 h, the water permeation rate decreased slightly (by 10% from its initial value), whereas the pristine PA membrane showed reduction in permeation rate of water by 40% from its initial value after 24 h. With active chlorine test at 20 ppm, it was found that due to the rapid deterioration of the PA membrane by chlorine, the pristine PA membrane rapidly dropped its salt rejection rate [140] and dramatically increased its water absorption rate after 100 h was also reported previously [141]. Moreover, CNF-PA membrane began to disintegrate at the same time with a lower pace compared to the PA membrane.

Hassan and group [142] developed Cu-terpyridine-modified oxidized cellulose nanofibers membrane (OXCNF-Cu-Tpy) and compared its performance with the TEMPO-oxidized cellulose nanofibrous membrane (OXCNF). Depending on the pressure applied during filtration, chemically modified OXCNF with Cu-Tpy groups boosted membrane’s pure water flux by 52 to 94% (0.5 and 1 MPa, respectively). Despite the fact that both OXCNF and OXCNF-Cu-Tpy membranes were highly efficient at removing suspended particles with the size range of (0.05–0.22 micron) from wastewater effluent, OXCNF-Cu-Tpy membrane showed a 30% higher flux rate than OXCNF membranes. Ref. [60] developed a nanofiber composite membrane with the help of corn stalks, by using it as the raw material. The team observed that the membrane was extremely successful at removing contaminants (diameter 1–100 nm) from waste water, and the retention rate of chromium (Cr(VI)) at pH = 11 was 80%, indicating that it can be utilized for short-term wastewater treatment and household water purification purposes.

The effect of cellulose nanofibers’ incorporation on cellulose acetate membrane’s morphology, water flux, and filtration performance was investigated by [143]. Due to variations in the remixing process rate during membrane development, an increase in CNF content resulted in a sponge-like shape. Porosity and pure water flux (40 L·m^−2^·h^−1^ for pure CA to 880 L·m^−2^ h for CA/CNF) improved as CNF content increased, and retentate turbidity was 11% greater after the separation procedure.

Hence, from the examination of various studies in this section, it was noted that the CNF incorporated membranes can considerably improve the performance of membranes in the reverse osmosis process. Table 3 presents the overview of the studies carried out using CNF-based membranes for the desalination and water treatment application. The different modified membranes, such as cellulose nanofibers, incorporated cellulose acetate membrane, 2,2,6,6-tetramethylpiperidine-1-oxyl radical (TEMPO)-oxidized CNF incorporated TFC membrane, Cu-terpyridine-modified cellulose nanofibers membrane, and TEMPO-oxidized cellulose nanofibrous membrane, and CNF modified TFC membranes can enhance the performance of membranes in the reverse osmosis process. There are also a lot of application for CNF-based membranes along with wastewater treatment.

## 7. Bacterial Nano Cellulose (BNC): Structure, Property, Wastewater, and Desalination Application

**Figure 9:**
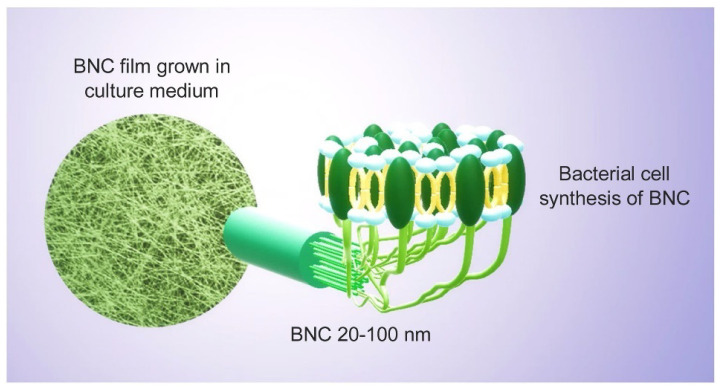
Representation of BNC synthesis. Reprinted with permission from Ref. [40]. Copyright 2020 Elsevier Ltd. All rights reserved.

Bacterial nanocellulose can be synthesized using a two-step process called polymerization and crystallization. Glucose residues polymerize in the bacterial cytoplasm to form β-1,4 glucan linear chains, which are secreted extracellularly. The generated chains are crystallized into microfibrils, which are subsequently consolidated to form a very pure three-dimensional (3D) porous network of entangled nanoribbons with a width of 20–60 nm [148]. This is because of its exceptional chemical and physical stabilities, greener synthetic approach, low manufacturing costs, hydrophilic nature, and good degradability. Due to the aforementioned reasons, bacterial nanocellulose is gaining increasing global interest, and hence scientists are focusing more studies on the BNC-based materials [149,150]. In many applications, the ultra-fine structure (as shown in Figure 9) of BNC outperforms plant cellulose in terms of stiffness 148], water interactive capacity, rate of polymerization, and active surface area [59].

**Figure 10:**
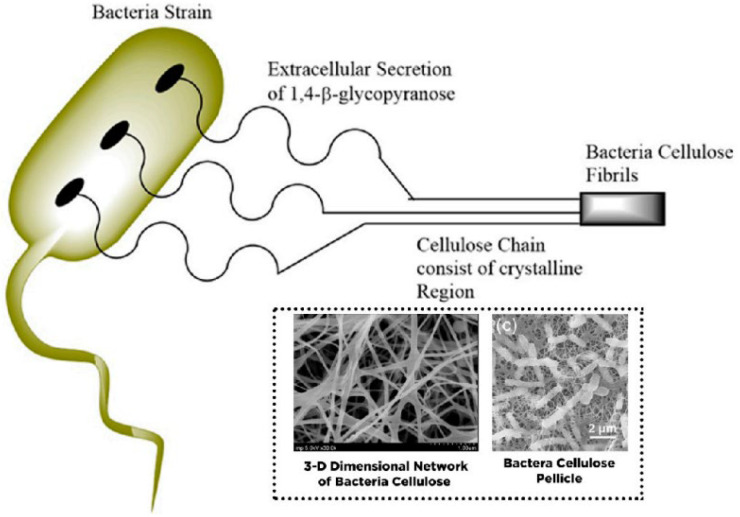
Diagrammatic representation of preparation of bacteria cellulose by means of extracellular secretion (SEM) images of three-dimensional network of bacteria cellulose, TEM images of BC pellicle [154].

The abundance of hydroxyl groups makes it easier for BNCs to functionalize or incorporate it with other reinforcing chemicals to give additional physical/chemical properties to the membranes [149], such as antibacterial properties [151,152]. As a result, BNC’s application areas are constantly expanding, including bioprocessing, biomedical, and pharmaceutical applications, food industry [153], wastewater treatment [153], and many more. Additionally, when compared to cellulose derivatives from plants, the BNC’s ability to be modified makes this material far superior. The bacteria strains are usually incubated in a nutrient-dense aqueous media and develop bacterial cellulose on the top layer (interface with air) as an exopolysaccharide. Here, the β-D-glucopyranose units are primarily present at the time of the development of cellulose molecules inside the bacteria cell [154]. The elementary fibril is discharged across the cellulose surface pores, which were additionally arranged as well as crystallized into microfibrils with twisting ribbons shape succeeded by pellicle development (Figure 10).

**Figure 11:**
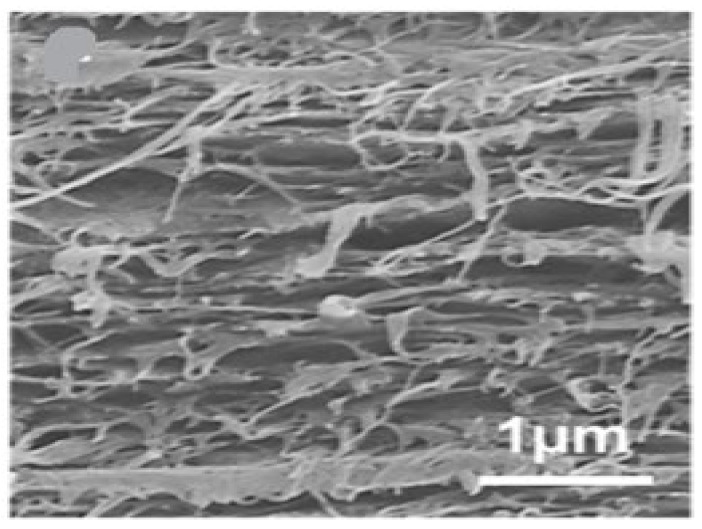
A cross-section of a pristine BNC membrane. Reprinted with permission from Ref. [155]. Copyright 2019 American Chemical Society.

Accounting for all the properties and application, the majority of researchers in the field of water desalination consider BNCs as a promising material and are continuously involved in the fabrication of many diverse membranes for enhancing the desalination performance of membranes [61]. Figure 11 is a cross-section of a pristine BNC membrane. BNCs are widely utilized in a variety of applications, but there has been little research on their use in membrane fabrication for desalination applications; therefore, we only mention a few bacterial nanocellulose based membrane applications in the following section.

### 7.1. Membrane Distillation

Membrane distillation is a technology which separates out the water in vapor form from saline water with higher rejection factors. Only vapor molecules are allowed to pass through a porous hydrophobic membrane in MD, and this process will be thermally driven membrane-based separation process [156]. The vapor pressure difference caused by the temperature gradient across the membrane surface is the driving force in the MD process. Separation efficiency strongly depends on the volatility of the separating component and also the structure of the porous membrane [157]. The bacterial nanocellulose has been proven to have good application in the development of membranes for the MD process. This section of the study presents certain recent studies performed for the preparation and testing of MD membranes developed using BNC in the MD application.

In a research work performed by Leitch and group [158], the team fabricated a model high porosity membrane and used it to investigate the performance on distillation. In this study, the researchers used unsupported bacterial nanocellulose aerogel membranes in direct contact membrane distillation (DCMD). The authors claimed that the membrane had lower bulk thermal conductivity, a larger porosity, greater conductivity, and thinner fibers as compared to any other MD material previously reported. The developed membrane material demonstrated much higher intrinsic membrane permeability and thermal efficiency than symmetric PVDF phase inversion membranes with lower porosity. This was confirmed after performing modeling and tests. Further improvement in MD flux was noted from the development of macroporous fibrous membranes with aerogel-like porosity and heat conductivity in thinner-film shapes.

**Figure 12:**
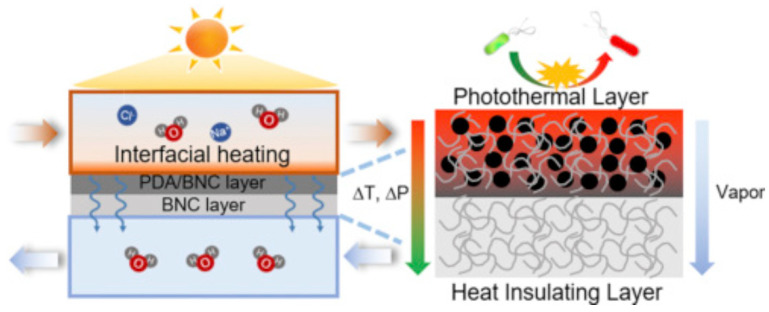
Schematic representation of solar-driven photothermal membrane distillation (MD) system. Reprinted with permission from Ref. [159]. Copyright 2020 Elsevier Ltd. All rights reserved.

Furthermore, Wu and group [159] fabricated an advanced membrane by making a bilateral structure composed of polydopamine (PDA) particles and bacterial nanocellulose together for photothermal membrane distillation with bactericidal capability. The developed membrane (Figure 12) showed a high solar energy-to-collected water efficiency of 68%, permeate flux of 1.0 kg m^−2^ h^−1^ under sun irradiation, an improved membrane porosity, and high salt rejection (>99.9%). The team also reported reduced conductive heat transfer, which increased the thermal efficiency of the membrane, and contributed to a stable hydrophobicity. Furthermore, the membrane exhibited good interfacial photothermal disinfection property to destroy germs when exposed to sunlight, allowing for easy cleaning, and thereby extending the membrane’s lifespan.

Hence, it was noted that the BNC incorporated membranes can considerably improve the performance of membranes in the membrane distillation process. The bacterial nanocellulose aerogel membranes and PDA/BNC membranes can enhance the performance of membranes in the MD process, and there are also a lot of application for BNC-based membranes along with wastewater treatment application.

### 7.2. Ultrafiltration

**Figure 13:**
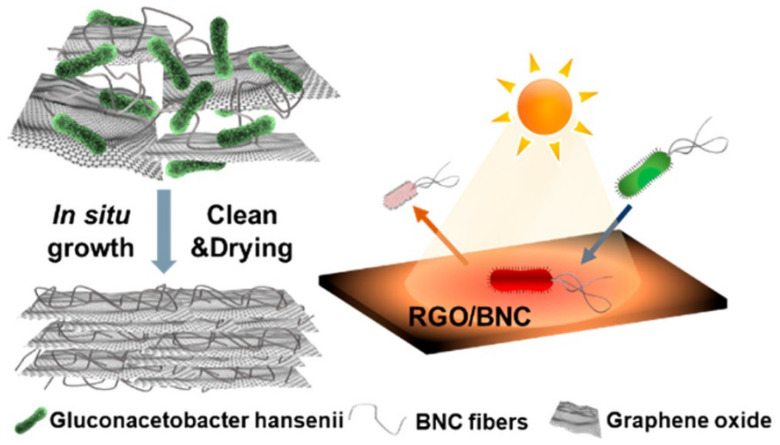
Schematic representation of UF membrane synthesis by using BNC fibers. Reprinted with permission from Ref. [155]. Copyright 2019 American Chemical Society.

The majority characteristics of the UF process are the same as membrane-based filtration, as the UF technique is aligned with the usage of semipermeable membrane. This section of the study presents certain recent studies carried out for the preparation and testing of advanced membranes developed using BNC in the ultrafiltration application. Jiang and group [155] used reduced graphene oxide and bacterial nanocellulose to fabricate an anti-biofouling UF membrane, as shown in Figure 13. The team tested the membrane stability under various conditions, and it was found that rGO/BNC membrane exhibited outstanding stability under environmentally relevant pH conditions, dynamic mechanical agitation/sonication, and even at high pressure. Over a 5 h-long flux test, the team compared the water flux of fabricated membrane with the commercial one, and it was noted that there was a two-fold increment in the water flux (52.6 ± 2.5 L/m^2^ h) for rGO/BNC membrane as compared with the water flux of commercial membrane (21.6 ± 0.8 L/m^2^ h).

Organic dyes are the most commonly seen contaminants present in industrial wastewater, and these materials are the most difficult to remove in any waste water treatment facility. Xu and group [160] fabricated an ultrafiltration membrane for the removal of organic dye based on bacterial nanocellulose with graphene oxide (GO) (while it was growing, essentially trapping GO in the membrane to make it stable and durable) and palladium (Pd) nanoparticles. The fabricated membrane shows outstanding performance by removing methylene orange (99.3%). The membrane was also tested for mixture of 4-nitrophenol and rhodamine 6G. The membranes showed a stable flux (33.1 L m^−2^ h^−1^) under 58 psi, over longer duration. Gholami and group [161] showed the membrane’s efficiency in heavy metal (Pd and Cd) and organic dye removal by using in situ entrenchment of polydopamine and bacterial nanocellulose particles. The membrane was further tested at several pH levels (4–7) and found to be quite stable.

Many researchers also fabricated BNC-based membranes for treating wastewater to make it proficient in desalination application [161] fabricated a membrane composed of bacterial nanocellulose and polydopamine materials. This membrane was tested for the removal of organic dyes (which are organic contaminants) and metal ions (lead and cadmium) present in the wastewater. It was found that the membrane was removing all the contaminants effectively.

Therefore, from the analysis of different studies, it was noted that the BNC-added membranes can considerably improve the performance of membranes in the ultrafiltration process. The Pd/GO/BNC membranes (organic dye and heavy metal removal), bacterial nanocellulose/polydopamine membranes (removal of organic contaminants and metal ions), and reduced graphene oxide/bacterial nanocellulose membranes can improve the performance of membranes in the ultrafiltration process, and there is also a huge application possibility for BNC-based membranes in desalination and wastewater treatment application, which need to be further explored. The novel design as well as the in situ inclusion of the membranes prepared in these studies presented a perception for obtaining advanced, ecofriendly, and fouling resistant membranes for water treatment application.

### 7.3. Other Application

For membrane-based water filtration application, Sijabat and group [162] developed nano cellulose derived from bacteria using Nangka banana peel media. The results confirmed the development of a potential membrane for an effective membrane filtration. The BNC colloidal solution was stable enough to be used in the manufacture of water filter catalytic membrane composites, according to the results obtained from electrophoretic light scattering (ELS) potential zeta absolute value (−11.39 mV). Moreover, a different study by the team fabricated an acetobacter xylinum-derived membrane for the application of PEG rejection. The team utilized β-chitin and deacetylated chitin sulfonates for solute separation. After performing the test, the team reported that all the membranes were performing exceptionally with respect to PEG50000 rejection, as each membrane contributed >85% rejection.

To avoid membrane fouling, pore blockage, and biofilm formation during the desalination process, a variety of chemically pretreatment processes are used these days, including chlorination, filtering, flocculation/sedimentation, and antiscalant dosage acidification [163]. The difficulty that has put these approaches on the back foot is their lack of reusability and chemical unitability. To solve this problem, researchers are looking into ecologically friendly materials, one of which is without a doubt nanocellulose.

Through a simple paper production procedure, Mautner and group [164] developed a bacterial nanocellulose based porous filter paper dispersed in low surface tension liquids and water for the removal of nanosized particles. When compared to traditional nano papers made from aqueous dispersions, nano papers made from organic solution had 40 times higher permeance due to a reduced paper density and high porosity. Despite their increased porosity, nano sheets feature pore diameters of 15–20 nm, which are similar to BC nano papers generated from aqueous dispersions, allowing for the size-exclusion removal of pollutants the size of viruses at high permeance.

Thus, overall, it was noted that the bacterial nanocellulose can be used effectively for the desalination as well as wastewater treatment application. Table 4 presents the overview of the studies carried out using BNC-based membranes for the desalination and water treatment application.

## 8. Future Perspectives

Nanocellulose has a lot of potential in the treatment of wastewater as well as in desalination. Nanocellulose is a one-of-a-kind solution in the field of wastewater treatment due to its mechanical strength, chemical stability, and resistance to environmental harshness. Researchers from all around the world have developed advanced membranes with promising results, but there has been little study on the application of BNCs and CNFs in the fabrication of desalination membrane. The majority of the study focuses on incorporating nanocellulose into the PA layer, but there is high potential to develop nanocellulose-based membranes by embedding this material in the support layer of the thin-film composite membranes, as well. The hydroxyl group of these nanocelluloses makes them appropriate candidates in the effort of membrane improvement. By considering this attribute of nanocellulose, there is a lot of scope for chemical alteration with naturally sound materials such as quantum dots and organic oxides.

Several recent studies have confirmed that the chemical modification of nanocellulose-based membranes is extremely significant for improving the membrane efficiency and the interactions between the contaminants and the membranes. The nanocellulose-based membranes with certain functionality can remove the required contaminants/ions present in water within a shorter time, thereby generating the fresh water efficiently. Therefore, the modification of membrane is anticipated to perform an important role in the forthcoming membrane development for desalination and water treatment applications.

It is recommended to have more studies based on developing membranes using the bacterial nanocellulose, cellulose nanocrystals, and cellulose nanofibers. Researchers should also consider cellulose extraction methods that are significantly more environmentally friendly, as well as the use of waste in this process.

## 9. Conclusions

As a sustainable and environmental-friendly material, the nanocellulose-based membranes are anticipated to extend its use to more applications shortly. The different forms of nanocellulose (CNF, CNC, or BNC) are able to be converted into the membrane itself or integrated into membranes as additives.

The results obtained from different studies on nanocellulose crystal inclusion, whether unmodified or modified, on the membrane have improved the desalination efficiency of the membranes. Surface modification can affect membrane performance, and the surface of CNC has many side hydroxyl groups, which allows for chemical modification, and this can affect membrane performance directly. The CNCs, when incorporated into the TFC membranes, showed improvement in the nanofiltration efficiency of the membrane, and these membranes are noted to have a big impact on the future development of high-performance NF membranes for water treatment. Moreover, it was noted that CNC-embedded TFC membranes showed higher permeate flux and salt rejection in RO testing. The CNC-based membranes also showed promising application in pervaporation. After running the CNC-modified pervaporation membrane, it was observed that the membrane showed an increment in the water flux with the factor of 3.

Moreover, the CNF-modified membranes are one of the best and cheapest options, as they exhibit high permeability, ultra-high water flux, better hydrophilicity, and good salt rejection. The surface modification of ultrafine cellulose nanofiber membranes by means of interfacial polymerization can develop ultrathin polymeric nanofiltration membranes of higher efficiency. The membrane becomes stronger and stiffer because of the strong bonding between PA matrix and CNF, and due to this, the matrix mobility decreases. CNF-PA membranes show great hydrophilicity, which is an important parameter for the nanocomposite membrane’s higher water permeability. The antifouling performance and chlorine resistance of the membranes will also be improved. Furthermore, considering the properties of BNCs, majority researchers in the field of water desalination consider BNCs as a promising material in desalination and water treatment, and are continuously involved in the fabrication of many diverse membranes for enhancing the desalination performance of membranes.

## Figures and Tables

**Table 1:** Description of different desalination technologies with the required pressures and membrane pore size [24,25,26,27,28,29,30].

Process	Reverse Osmosis	Forward Osmosis	Nanofiltration	Ultrafiltration	Pervaporation	Membrane Distillation
Membrane Pore diameter	<0.5 nm	0.50–0.60 nm	<2.0 nm	0.001 –0.05 mm	0.2 to 0.6 nm	100 Å and 1 μm.
Required pressure (bar)	High pressure	Mild pressure	High pressure	Low pressure	Low pressure	-

**Table 2:** The overview of the studies carried out using CNC-based membranes for the desalination and water treatment application.

Cellulose Material	Cellulose Raw Material	Method of Synthesis	Application	Target	Foulant	Removal Efficiency	Reference
CNC incorporated membrane	Filter paper	Acidic hydrolysis	Osmotically derived FO	NaCl	BSA	Salt rejection: 89.79%	[120]
PES/CNC/APTES membrane	-	-	adsorption and ion removal	Cu ion and Red-16	-	90% Cu ion 99% Red-16	[121]
CNCs/PMVEMA/PEG aerogel	Wood pulp	-	adsorption	cation methylene blue dye	-	116.2 mg g^−1^	[122]
CNC membrane	Kapok fiber		adsorption	Methyl blue		85% for 5 mg/L MB	
PVDF-HFP/CNC membrane	cellulose KimWipes	Acid Hydrolysis	MD	NaCl	-	Salt rejection 99% and Water flux 10.2–11.5 Lh^−1^ m^−2^	[123]
GO-CNC/PVDF membrane	Hawp04700-Merck Millipore cellulose membrane	Acid Hydrolysis	Membrane bioreactor	Municipal waste water	BSA	2.8 times less fouling	[124]
CNC/AgNPs pebble	Cellulose powder	Acid hydrolysis	Adsorption	Pb and Cr ion		99.48% Pb(II) and 98.30% Cr(III)	[125]
CNC/UF	Cotton linter	Hydrolysis	UF	vitamin B12, Blue Dextran, and β-lactoglobulin		2.6, 4.1, and 26%	[126]
PSF/CNC	-	Hydrolysis	UF	Petroleum effluent	Oil particles	94.4%	[127]

**Table 3:** The overview of the studies carried out using CNF-based membranes for the desalination and water treatment application.

Cellulose Material	Cellulose Raw Material	Method of Synthesis	Application	Target	Foulant	Removal Efficiency	Reference
CNF based filter paper	*Cladophora* sp. algae	-	Size-exclusion filtration	xenotrpic murine leukemia virus	-	5.25log xMuLV	[144]
CNF/TFN	Wood pulp	TEMPO/NaBr/NaClO method	UF	oil/water emulsion	sodium alginate	96.3%	[145]
Titanate-bismuth oxide/CNF	Bamboo pulp	TEMPO oxidation	Adsorption	Cs^+^ and I^−^ ion	Industrial oil	is <80%	[146]
Fe_3_O_4_/CNF magnetic membrane	Sugarcane pulp	Hydrolysis	Catalytic dye removal	RhB	-	94.9%	[23]
UF/PSF/CNF	-	-	UF	Blue dextran		99%	[147]

**Table 4:** The overview of the studies carried out using BNC-based membranes for the desalination and water treatment application.

Cellulose Material	Cellulose Raw Material	Method of Synthesis	Application	Target	Foulant	Removal Efficiency	Reference
BNC/Ag	BNC	-	Antimicrobial activity	E. coli and S. aureus	-	Inhibitory rate 99.4% and 98.4%	[165]
BNC based membrane	Banana Peel	Fermentation with Gluconacetobacter xylinus bacteria	Filtration	*-*	-	-	[162]
Pd/GO/Bacterial NC	Pd, Graphene oxide and BNC	Fermentation	Filtration	Gold Nanoparticles		99.7%	[164]
BNC based nanopaper	Bacterial Cellulose	-	UF	MO		99.3% over different concentration of MO	[160]
rGO/BNC	Gluconacetobacter hansenii	In situ incorporation	UF	Au, E. coli		97%	[166]
PDA/BNC	Gluconacetobacter hansenii	In situ incorporation	NF	R6G, MO, MB		Avg. 89%	[161]

## Data Availability

Not applicable.
